# Early-Life Microbiota Modulation and Neurodevelopment in Infants: A Systematic Review and Meta-Analysis of Randomized Controlled Trials

**DOI:** 10.3390/cells15070638

**Published:** 2026-04-01

**Authors:** Salvatore Michele Carnazzo, Fabio Allia, Alice Foti, Marilena Briglia, Marcello Maida, Adriana Carol Eleonora Graziano, Andrea Domenico Praticò

**Affiliations:** Department of Medicine and Surgery, University of Enna “Kore”, 94100 Enna, Italy; fabio.allia@unikorestudent.it (F.A.); marilena.briglia@unikore.it (M.B.); marcello.maida@unikore.it (M.M.); adriana.graziano@unikore.it (A.C.E.G.); andrea.pratico@unikore.it (A.D.P.)

**Keywords:** gut microbiota, probiotics, prebiotics, synbiotics, infant neurodevelopment, gut–brain axis, early-life microbiota modulation, randomized controlled trials

## Abstract

Changes in microbial composition during early infancy by various factors (mode of delivery, nutritional practices, antibiotic usage, and environmental influences) have been correlated with observable variances in cognitive abilities, temperament, stress response, and the predisposition to neurodevelopmental disorders. Consequently, microbiota-targeted interventions such as probiotics, prebiotics, and synbiotics are being explored as avenues to enrich beneficial microbial taxa, enhance short-chain fatty acid production, fortify mucosal immunity, and mitigate inflammatory responses during these critical periods. Preclinical research, primarily in experimental animal models, has demonstrated a causal link between microbiota composition and developmental processes such as myelination, synaptic plasticity, and socio-emotional behaviors, whereas human evidence remains largely associative and heterogeneous. A notable gap exists in the current literature, which typically centers on gastrointestinal, psychiatric, or preterm outcomes, without a focused investigation into neurodevelopmental assessments within the first three years. To bridge this gap, we conducted a systematic review and meta-analysis of randomized controlled trials assessing the impact of probiotics, prebiotics, and synbiotics on neurodevelopment and behavior in infants aged 0–36 months. Our primary objective was to establish whether microbiota-targeted strategies confer discernible neurodevelopmental benefits, alongside elucidating the mechanisms underpinning the relationship between microbial modulation and early brain development.

## 1. Introduction

The first years of life constitute a critical window of rapid brain growth, synaptogenesis, and neuroplasticity, during which early environmental exposures can shape long-term cognitive, motor, and socio-emotional trajectories [1].

Within this context, the developing gut microbiota has emerged as a potential modulator of neurodevelopment through the bidirectional gut–brain axis, which integrates microbial metabolites, immune and endocrine signaling, and vagal pathways [2].

Marked shifts in microbial composition occur across early infancy and are influenced by delivery mode, feeding practices, antibiotic exposure, and environmental factors [3]. Perturbations of these early colonization patterns have been associated with differences in cognition, temperament, stress responsivity, and later neurodevelopmental disorders [4,5].

Microbiota-targeted interventions such as probiotics, prebiotics, and synbiotics have therefore been proposed as strategies to promote beneficial taxa, modulate short-chain fatty acid production, strengthen mucosal immunity, and attenuate inflammatory signaling during sensitive developmental periods [6].

Preclinical studies in germ-free and antibiotic-exposed models support a causal role of microbiota composition in shaping myelination, synaptic plasticity, and socio-emotional behaviors [7]. However, it is important to note that these causal relationships have been demonstrated primarily in experimental animal models, while evidence from human studies remains largely associative. Translational human evidence has similarly suggested associations between early-life microbiota profiles and cognitive or behavioral trajectories, although findings remain heterogeneous [8].

Despite strong biological plausibility, it remains unclear whether early-life microbiota modulation produces measurable neurodevelopmental benefits. Randomized controlled trials (RCTs) in diverse infant populations have reported mixed or null effects across developmental domains [9,10]. Existing reviews have focused on gastrointestinal outcomes, psychiatric symptoms, or preterm morbidity rather than validated neurodevelopmental assessments within the first three years [11]. Unlike previous systematic reviews, the present study specifically focuses on validated neurodevelopmental assessments during the first three years of life and integrates clinical neurodevelopmental outcomes with mechanistic biomarkers related to gut–brain axis signaling.

**Figure 1:**
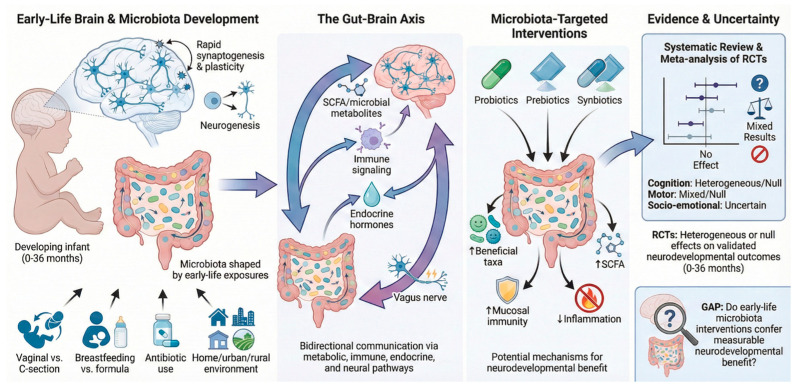
Overview of potential beneficial (pros) and adverse or uncertain (cons) effects of microbiota-targeted interventions on the gut–brain axis in early life. The figure schematically illustrates the main biological pathways linking gut microbiota modulation (via probiotics, prebiotics, and synbiotics) to neurodevelopmental processes, including immune signaling, microbial metabolite production (e.g., short-chain fatty acids), and neuroendocrine pathways. Arrows indicate proposed mechanistic links, while opposing elements summarize potential benefits and limitations reported in the literature. This figure was created using FigureLab (an AI-assisted graphical tool, latest available version at the time of use) and subsequently reviewed, edited, and validated by the authors to ensure scientific accuracy and consistency with the evidence presented in the manuscript.

To address this gap, we conducted a systematic review and meta-analysis of RCTs evaluating probiotic, prebiotic, or synbiotic interventions in infants aged 0–36 months (Figure 1). Our aim was to determine whether early microbiota-targeted strategies confer measurable neurodevelopmental or behavioral benefits and to evaluate whether these interventions produce clinically meaningful improvements in standardized neurodevelopmental assessments during the first three years of life.

## 2. Materials and Methods

### 2.1. Study Design and Registration

This systematic review and meta-analysis were conducted according to PRISMA 2020 guidelines [12]. The completed PRISMA 2020 checklist is provided as Supplementary Material (Supplementary Table S1).

The protocol was prospectively registered in the PROSPERO international database (registration number CRD420251207391), under the title “Early-life microbiota modulation and neurodevelopment: a systematic review and meta-analysis of randomized controlled trials on probiotics, prebiotics, and synbiotics in infants under 36 months” [13]. The predefined PICO framework, eligibility criteria, and statistical analysis plan were established a priori and guided all stages of the review, according to the methodological structure described in the protocol.

### 2.2. Eligibility Criteria

#### Population

We included randomized controlled trials enrolling human infants aged 0–36 months, born at term or preterm. Studies involving healthy infants or infants with mild conditions not directly affecting brain development (e.g., infantile colic) were eligible. We excluded studies enrolling infants with:

1. Major congenital anomalies or chromosomal syndromes;
2. Severe acquired neurological injury (e.g., intraventricular hemorrhage grade III–IV, hypoxic–ischemic encephalopathy);
3. Need for long-term parenteral nutrition or gastrointestinal surgery.

### 2.3. Interventions and Comparators

Eligible interventions included probiotics, prebiotics, and synbiotics, administered orally in any formulation (drops, powder, fortified milk/formula), dose, strain, or duration. Comparators included:

1. Placebo;
2. Standard formula;
3. No intervention.

### 2.4. Outcomes

#### 2.4.1. Primary Outcomes

Validated neurodevelopmental assessments performed before 36 months, including:

1. global composite scores (Bayley Scales, Griffiths, Mullen Early Learning Composite),
2. domain-specific outcomes (cognitive, language, motor, social-emotional, adaptive functioning), as prespecified in the protocol

#### 2.4.2. Secondary Outcomes

1. Behavioral and sleep outcomes (e.g., colic severity, crying duration, validated sleep measures);
2. Gut–brain axis biomarkers (gut microbiota composition, SCFAs, immune markers including sIgA, calprotectin, cytokines);
3. Stress physiology (salivary cortisol);
4. Growth parameters;
5. Adverse events.

Studies reporting only non-validated parental reports (e.g., “crying more/less”, stool frequency without validated metrics) without standardized neurodevelopmental or physiological outcomes were excluded.

### 2.5. Study Design

We included parallel-group randomized controlled trials, individually randomized or cluster-randomized. Crossover RCTs were included using first-period data only.

We excluded non-randomized, observational, quasi-experimental and uncontrolled trials.

### 2.6. Language and Publication Data

Only full-text English-language articles, published from 2010 onwards, were included.

This restriction was applied to ensure methodological comparability with contemporary studies, reflecting the widespread adoption of modern microbiome profiling technologies (such as 16S rRNA sequencing and metagenomics) and updated standardized neurodevelopmental assessment tools used in recent randomized trials.

### 2.7. Information Sources and Search Strategy

A comprehensive search was conducted in PubMed, Scopus, and Web of Science from database inception to October 2025.

Search strings combined terms for population, intervention, neurodevelopmental outcomes, and randomized trial design. An example search strategy used in PubMed was as follows: (infant OR neonate OR newborn) AND (probiotic OR prebiotic OR synbiotic OR microbiota) AND (neurodevelopment OR cognition OR behavior OR Bayley) AND (randomized controlled trial OR RCT).

Equivalent Boolean search strings adapted to database-specific syntax were applied in Scopus and Web of Science.

The complete Boolean search strategy is documented in the protocol and reproduced in the extracted query set. Additional sources included:

1. Manual screening of reference lists of eligible studies;
2. Searches of ClinicalTrials.gov and WHO ICTRP trial registries.

No restrictions were applied for setting, country, or funding source.

### 2.8. Study Selection

All records were imported into Rayyan (Qatar Computing Research Institute, Doha, Qatar; web-based platform, accessed in 2025) for deduplication and screening.

Two reviewers independently screened titles and abstracts, followed by full-text assessment against the predefined eligibility criteria.

**Figure 2:**
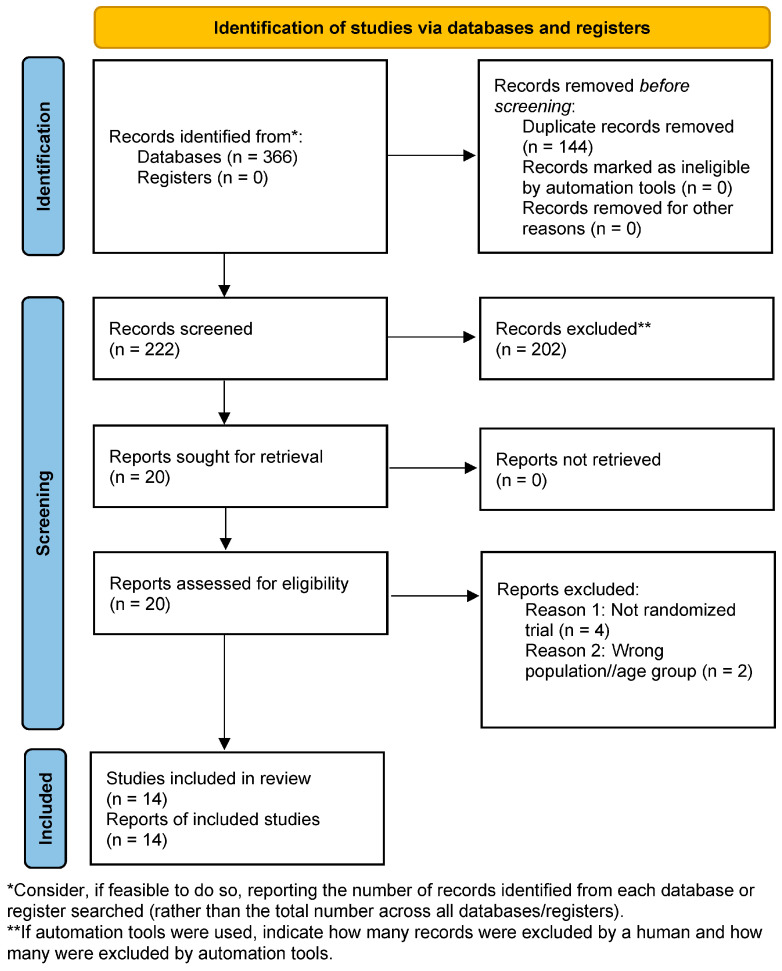
PRISMA 2020 flow diagram illustrating the study selection process for randomized controlled trials evaluating microbiota-targeted interventions and neurodevelopmental outcomes in infants aged 0–36 months.

Disagreements were resolved through discussion; no arbitration was ultimately required. Reasons for exclusion at the full-text stage were documented and reported in a PRISMA 2020 flow diagram (Figure 2).

### 2.9. Data Extraction

Data were extracted independently by two reviewers into a standardized spreadsheet. Extracted variables included the following:

1. Study characteristics (country, year, design, sample size);
2. Participant characteristics (age, gestational age, feeding modality);
3. Intervention details (strain, dose, duration, formulation);
4. Comparator characteristics;
5. Neurodevelopmental outcomes (tool, domain, scoring method, timepoint, mean ± SD or event counts);
6. Mechanistic biomarkers (microbiota composition, SCFAs, sIgA, inflammatory cytokines, antimicrobial peptides, cortisol);
7. Gastrointestinal and behavioral outcomes;
8. Adverse events;
9. Data required for meta-analysis (group means, SDs, N; conversions from medians/IQRs when applicable).

When studies reported values only graphically, numerical estimates were extracted using validated digital extraction tools. Two reviewers independently digitized the data and cross-checked extracted values to minimize measurement error, with discrepancies resolved by consensus.

### 2.10. Risk of Bias Assessment

Risk of bias was assessed using the Cochrane Risk of Bias 2.0 tool, covering the following [14]:

1. Randomization process;
2. Deviations from intended interventions;
3. Missing outcome data;
4. Measurement of outcome;
5. Selection of reported results.

Each study was classified as low risk, some concerns, or high risk. Assessments were performed independently and resolved by consensus.

### 2.11. Data Synthesis and Statistical Analysis

#### 2.11.1. Meta-Analysis

Meta-analysis was performed only when ≥2 studies evaluated the same outcome using the same validated instrument at comparable ages, following Cochrane guidance.

Because only Bayley-III outcomes were reported homogeneously, quantitative pooling was conducted for:

1. Bayley-III Cognitive Composite;
2. Bayley-III Language Composite;
3. Bayley-III Motor Composite.

Continuous outcomes were synthesized using mean differences (MD) with 95% confidence intervals. Random-effects models (DerSimonian–Laird) were applied because clinical heterogeneity across studies was expected, including differences in infant populations, probiotic strains, intervention duration, and study design. All other outcomes (ASQ, BSID-II, MSEL, behavioral outcomes, biomarkers) were analyzed narratively due to heterogeneity in scales, reporting units, or timepoints.

##### Heterogeneity and Publication Bias

Statistical heterogeneity was assessed using I^2^ statistics and χ^2^ tests.

Publication bias assessment (funnel plots, Egger’s test) was planned for analyses including ≥10 studies; this threshold was not reached.

##### Sensitivity Analyses

Pre-planned sensitivity analyses (exclusion of high-risk-of-bias studies; exclusion of follow-up <8 weeks for neurodevelopmental outcomes) were not feasible due to the small number of studies contributing to each meta-analysis.

##### Software

Statistical analyses were conducted using Review Manager (RevMan), version 5.4 (The Cochrane Collaboration, London, UK).

##### Certainty of Evidence

The certainty of evidence for each primary outcome was assessed using the GRADE approach [15], evaluating the following:

1. Risk of bias;
2. Inconsistency;
3. Indirectness;
4. Imprecision;
5. Publication bias.

## 3. Results

### 3.1. Characteristics of Included Studies

Fourteen studies derived from randomized controlled trials met the inclusion criteria. Of these, seven randomized trials enrolling 733 infants in probiotic or synbiotic intervention arms and 735 in control groups contributed quantitative data to the primary analyses [16,17,18,19,20,21,22,23,24,25,26,27,28,29].

Studies were conducted across Europe, Asia, and Oceania between 2011 and 2025. Five trials used a double-blind, placebo-controlled design, one employed a cluster-randomized community-based approach, and one evaluated a non-blinded probiotic-enriched formula. Populations consisted primarily of healthy term infants, with one large trial enrolling late preterm infants [17], and one including term infants with colic [16]. All interventions were administered postnatally in early infancy; no trial investigated maternal prenatal supplementation. Sample sizes ranged from 27 to 863 participants, contributing to variability in statistical power. Interventions differed in microbial strains, dose, and duration. Frequently used strains included *Lactobacillus plantarum* HEAL9, *Lactobacillus reuteri* DSM 17938, *Bifidobacterium animalis* subsp. *lactis* BB-12, and *Bifidobacterium breve* M-16V, as well as several multistrain synbiotic formulations.

Daily doses ranged from approximately 10^8^ to 10^10^ CFU, and treatment duration varied from 3 to 12 months. Comparators were predominantly placebo or standard formula, except for one modified-formula trial without live microbes that was retained due to relevant neurodevelopmental endpoints [18]. Neurodevelopmental assessments included the Bayley Scales of Infant Development, Third Edition (Bayley-III), the Ages & Stages Questionnaire (ASQ), and the Mullen Scales of Early Learning (MSEL).

Follow-up ranged from 3 to 18 months, with the longest evaluation at 18 months corrected age [17]. Risk-of-bias assessment indicated two studies at low risk and four with some concerns, largely due to incomplete variance reporting or unclear allocation concealment; one cluster-randomized trial lacked sufficient detail for full appraisal.

### 3.2. Outcomes Assessed

Neurodevelopmental outcomes were categorized a priori into five domains: cognitive, language, motor, behavioral/socio-emotional, and mechanistic. Across trials, outcomes were measured using standardized clinician-administered tools and validated parent-reported questionnaires. Data were reported as continuous standardized scores (Bayley-III composite scores, MSEL T-scores, ASQ domain scores) or as categorical classifications based on predefined impairment thresholds, including developmental cut-offs, neurosensory impairment, and cerebral palsy. Assessment timepoints ranged from early infancy (as early as 16 weeks in term infants) to preschool age in preterm cohorts (30–54 months), allowing characterization of both early developmental status and emerging longer-term trajectories.

#### 3.2.1. Cognitive Outcomes

Cognitive development was assessed using the Bayley Scales of Infant Development and the Mullen Scales of Early Learning (MSEL). Two probiotic trials in extremely or very preterm infants reported Bayley-III Cognitive Composite Scores at 24 months [19] and 30 months [17]. Earlier versions of the Bayley scales contributed additional cognitive endpoints through the BSID-II Mental Development Index (MDI), reported either as continuous scores or as the proportion of infants with significant delay (MDI < 70) [16,21].

**Figure 3:**
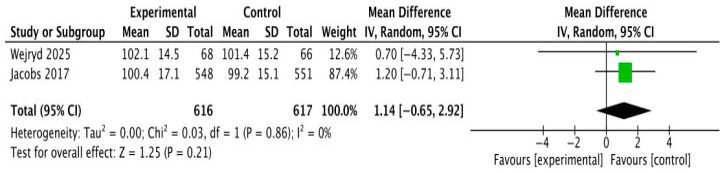
Forest plot of Bayley-III Cognitive Composite scores comparing microbiota-targeted interventions with control groups (Jacobs 2017 [17]; Wejryd 2025 [19]). Effect sizes are expressed as mean differences (MD) with 95% confidence intervals using a random-effects model. Green squares represent individual study estimates (with size proportional to study weight), and the black diamond indicates the pooled overall effect size.

Longer-term outcomes were available from a UK preterm cohort assessed at 54 months with the MSEL Early Learning Composite and Visual Reception domain (Agrawal 2020) [22]. Parent-reported cognitive indicators, including the ASQ-3 Problem-Solving domain, were reported in two studies [18,20]. Meta-analysis of the two studies providing continuous Bayley-III cognitive scores demonstrated no significant effect of probiotic supplementation (MD 1.14, 95% CI −0.65 to 2.92; I^2^ = 0%, Figure 3). However, the meta-analysis for this outcome included only two studies, which limits the statistical power and precision of the pooled estimate and should be considered when interpreting the results. Across individual trials, cognitive performance remained within age-expected ranges, and no study reported clinically meaningful probiotic-associated improvement. Binary outcomes of cognitive impairment similarly showed no group differences [17]. Overall, evidence consistently indicates that early-life probiotic or microbiota-targeted interventions do not improve cognitive development in infancy or early childhood.

#### 3.2.2. Language Outcomes

**Figure 4:**
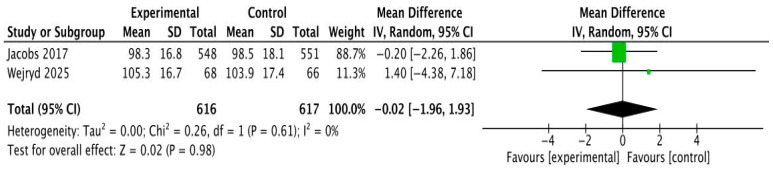
Forest plot of Bayley-III Language Composite scores comparing microbiota-targeted interventions with control groups (Jacobs 2017 [17]; Wejryd 2025 [19]). Effect sizes are expressed as mean differences (MD) with 95% confidence intervals using a random-effects model. Green squares represent individual study estimates (with size proportional to study weight), and the black diamond represents the pooled effect estimate.

Language development was assessed using both clinician-administered tools and parent-reported measures. The primary standardized instrument was the Bayley-III Language Composite, incorporating receptive and expressive communication domains. This outcome was reported in extremely preterm infants at 24 months [19] and in very preterm infants at 30 months [17]. A pooled analysis showed no effect on language development: MD = −0.02 (95% CI −1.96 to 1.93), I^2^ = 0% (Figure 4). As only two trials contributed data to this meta-analysis, the limited number of studies reduces statistical power and the certainty of this estimate.

Preschool-age assessments in the trial by Agrawal et al. [22] included the MSEL Receptive Language and Expressive Language T-scores, both reported as medians (IQR) and showing no group differences. Parent-reported communication outcomes were available from the ASQ-3 Communication domain in C-section born infants [20] and formula-fed infants [18], with no significant differences in either continuous or categorical outcomes.

Across standardized and parent-reported measures, early-life probiotics and related nutritional interventions did not influence language development, with results consistently null across cohorts and ages.

#### 3.2.3. Motor Outcomes

**Figure 5:**
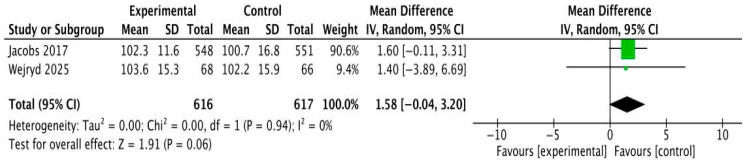
Forest plot of Bayley-III Motor Composite scores comparing microbiota-targeted interventions with control groups (Jacobs 2017 [17]; Wejryd 2025 [19]). Effect sizes are expressed as mean differences (MD) with 95% confidence intervals using a random-effects model. Green squares represent individual study estimates (with size proportional to study weight), and the black diamond represents the pooled effect estimate.

Motor development was captured using Bayley-III Motor Composite Scores (fine and gross motor performance) from Wejryd 2025 and Jacobs 2017 [17,19]. Both individual studies reported comparable performance between intervention and control groups. The pooled analysis indicated a non-significant trend favoring the probiotic group, but without statistical evidence of benefit: MD = 1.58 (95% CI −0.04 to 3.20), *p* = 0.06, I^2^ = 0% (Figure 5). This pooled estimate was also derived from only two studies, which restricts the robustness of the statistical inference. Additional motor outcomes from earlier Bayley editions included the BSID-II Psychomotor Development Index (PDI) [16,21], reported as continuous scores and as the proportion with PDI < 70, all showing no differences.

Preschool assessments using the MSEL Fine Motor domain [22] also demonstrated no effect. Parent-reported outcomes were captured by the ASQ-3 Fine Motor and Gross Motor domains [18,20]. Categorical thresholds (above/close/below cut-off) and continuous scores were consistently similar across groups.

Clinical motor outcomes, including cerebral palsy and motor impairment thresholds assessed by MABC-2 [17], showed no significant differences. Despite a borderline trend in pooled Bayley motor scores, the overall body of evidence demonstrates no robust or clinically significant impact of probiotic supplementation on motor development.

#### 3.2.4. Behavioral, Emotional, and Social Outcomes

Behavioral and socio-emotional development was less extensively assessed. Measures were primarily derived from the ASQ-3 Personal-Social domain [18,20]. Both continuous and categorical assessments at different timepoints reported no significant differences between intervention and control groups. In very preterm infants, broader functional outcomes, including major neurosensory impairment, survival without major neurosensory impairment, hearing impairment, visual impairment, and cerebral palsy, were assessed through clinical evaluation [16,17].

All results indicated no probiotic-associated differences. No study used validated behavioral psychopathology tools such as the CBCL, SDQ, or ITSEA. Evidence across all available socio-emotional and neurosensory outcomes shows no measurable effect of probiotic supplementation.

### 3.3. Overview of Secondary Outcomes

Across the included trials, secondary outcomes encompassed infant crying and fussing, sleep duration and quality, gastrointestinal symptoms and stool characteristics, anthropometric measures, parental quality of life and perceived child health, and a small set of mechanistic outcomes related to gut microbiota. These endpoints were assessed using a combination of parent-reported diaries and visual analogue scales (VAS), structured symptom questionnaires, and standard clinical measurements (e.g., weight, length, head circumference). Secondary outcomes were reported both as continuous variables (e.g. minutes of crying per day, hours of sleep, stool frequency, VAS scores, fecal Bifidobacteria %) and as categorical or percentage-based endpoints (e.g., proportion of infants with ≥25% reduction in crying, presence of diarrhea, watery/soft stools, recurrent night-time crying, parental concern about child health). Follow-up periods for these outcomes ranged from a few weeks in colic and formula-tolerance trials to several months in studies of C-section-born infants or preterm populations.

No secondary outcome was consistently reported by a sufficient number of homogeneous studies to allow meta-analysis; therefore, all secondary outcomes were synthesized narratively.

### 3.4. Crying and Fussing

Across trials in term or otherwise healthy infants, particularly those with colic, crying and fussing were assessed using both continuous and categorical measures. Continuous outcomes from Chen 2021 [23] and additional colic-focused studies (Turco 2021; Nocerino 2020; Gerasimov 2018 [24,25,26]) indicated lower daily crying time or fewer episodes among infants receiving probiotics or microbiota-targeted formulas, although heterogeneity in measurement tools and reporting limited comparability. Categorical responder analyses similarly suggested higher proportions of infants achieving clinically meaningful reductions in crying or fussing (e.g., ≥25% reduction), with weaker or inconsistent effects at stricter thresholds (e.g., ≥50%). Some studies also reported lower rates of intense crying or recurrent night-time crying in the intervention group, although incomplete data in certain trials restricted quantitative interpretation.

Overall, both continuous and categorical outcomes suggest that probiotics may modestly reduce crying and fussing in colicky infants, but variability in definitions, thresholds, and reporting limits the certainty of these findings.

### 3.5. Sleep Outcomes

Sleep was assessed in several trials using both continuous and categorical measures. Continuous outcomes from Chen 2021, Ren 2022, Nocerino 2020, and Gerasimov 2018 included daily sleep duration recorded in parental diaries (minutes or hours per day) [20,23,25,26]. Across studies, no consistent or statistically significant differences were observed between probiotic and control groups, with only small numerical trends that were not sustained across timepoints. Categorical sleep outcomes were reported less frequently and included parental ratings of sleep quality or sleep-related VAS scores (e.g., Turco 2021) [24], but these also showed no reproducible group differences.

Overall, available evidence indicates that probiotic supplementation does not materially influence sleep duration or sleep-related behavior in early infancy, although subtle effects on sleep quality cannot be excluded based on the limited and heterogeneous data.

### 3.6. Gastrointestinal Outcomes

Gastrointestinal outcomes were reported across several trials using both continuous and categorical measures. Continuous endpoints included stool frequency, timing of first stool, and other indicators of gastrointestinal motility. In the Wejryd ELBW cohort [19], day of first stool and the number of stools during the early postnatal period were recorded, while Ren 2022 and Nocerino 2020 measured daily stool frequency in probiotic versus control groups [20,25]; across these studies, stool frequency was generally similar between groups, with no consistent evidence of increased bowel movements beyond expected maturational changes. In some formula-based interventions, modest differences in stool pattern or consistency were noted, although these were typically reported using categorical classifications. Categorical gastrointestinal outcomes included diarrhea, abdominal pain, constipation, watery or soft stools, stool consistency categories, and broader feeding-tolerance indices. Ren 2022 reported proportions of infants with abdominal pain and constipation [20], while Nocerino 2020 and Turco 2021 compared distributions of stool consistency (e.g., watery stools, soft stools) [24,25].

Some interventions were associated with higher rates of soft stools or lower rates of watery stools or diarrhea, whereas other findings were small, inconsistent, or not replicated across studies. Gerasimov 2018 observed lower diarrhea rates in the intervention group, but composite gastrointestinal symptom scores generally showed no clear between-group differences [26].

### 3.7. Anthropometric Outcomes

Anthropometric secondary outcomes included weight, length, and head circumference at various follow-up timepoints. These measures were reported in Firmansyah 2011 (length and head circumference at follow-up in the context of gastrointestinal tolerance and growth) and Ren 2022 (weight and head circumference in infants supplemented with *L. paracasei* versus placebo) [20,27]. Across all trials, anthropometric indices were similar between intervention and control groups, with no indication of growth impairment or acceleration associated with probiotic or microbiome-targeted products. Where differences were tested formally, they were typically non-significant, and the direction of effect was coded as “no difference” in the extraction sheet.

Thus, secondary anthropometric data support the conclusion that probiotic or microbiome-oriented interventions do not materially affect short-term growth parameters in the populations studied.

### 3.8. Parental Quality of Life and Psychological Scales

Several studies assessed parental wellbeing and the perceived impact of infant symptoms using validated questionnaires and VASs. In Chen 2021 [23], parent health-related quality of life (HRQoL) was measured across domains of physical, emotional, and social functioning, all treated as continuous scores. These outcomes were generally coded as “higher = better”, and mean HRQoL scores tended to favor the probiotic group, suggesting modest improvement in parental functioning. In Turco 2021 [24], maternal and paternal VAS scores captured quality of life, perceived colic severity, and sleep quality. Several of these VAS outcomes showed favorable ratings for the probiotic or synbiotic intervention, particularly for colic severity and aspects of parental wellbeing, with some comparisons indicating clearly better scores or trends in the intervention group. Overall, continuous parental QoL outcomes suggest that reductions in infant symptoms in some colic-oriented trials may translate into modest improvements in parental quality of life and perceived burden, although effect sizes are inconsistently reported and cannot be pooled.

### 3.9. Appetite and Behavior

Several categorical outcomes reflected broader behavioral or comfort-related domains, such as appetite, quiet/general temperament, or composite GI-behavioral symptom scores. These were typically expressed as percentages of infants with a given symptom category or level of parental concern. Across trials, no consistent pattern emerged, indicating that probiotics or microbiome-targeted products systematically improve appetite or general behavioral profiles beyond changes already accounted for by crying/fussing or GI outcomes. Where differences were observed, they tended to be modest and often not supported by detailed statistical reporting.

### 3.10. Parental Concern and Global Impression

Parental perceptions of child health and symptom burden were captured categorically in some studies. In Ren 2022, parental concern for the child’s health was assessed as a percentage-based outcome [20]. Group differences were small and not clearly in favor of the probiotic group when adjusted or interpreted in the context of other child outcomes. Some studies also used categorical summaries of VASs or threshold-based classifications (e.g., presence vs absence of substantial concern), but these were infrequently reported and heterogeneous in definition. Overall, categorical parental concern outcomes suggest no consistent probiotic-associated reduction in parental worry or perceived child health problems, beyond the modest improvements in QoL captured by continuous scales in a subset of trials.

### 3.11. Overview of Mechanistic Outcomes

Eight randomized controlled trials reported at least one mechanistic biomarker related to gut microbiota composition or host physiological responses [18,21,23,24,25,27,28,29]. Reported biomarkers spanned multiple domains, including gut microbial composition (e.g., Bifidobacteria, Lactobacillus/Enterococcus, Enterobacteriaceae, Bacteroides/Prevotella, Clostridial clusters, total bacterial load), short-chain fatty acids (SCFAs), mucosal immunity (secretory IgA), antimicrobial peptides (LL-37, HBD-2), cytokines and inflammatory mediators, intestinal inflammation and permeability (calprotectin, α1-antitrypsin), gut environmental parameters (fecal pH), and stress physiology (salivary cortisol).

Substantial heterogeneity was evident across studies with respect to units of measurement (e.g., log10 CFU/g, relative abundance percentages, mg/g stool, mM, ng/g, nmol/L), assay methodologies (culture-based methods, qPCR, 16S rRNA profiling, ELISA, multiplex cytokine panels), and timing of sample collection, which ranged from the neonatal period to 12–16 months of age. Reporting formats also varied (means with SD, medians with IQR, or graphical estimates only), further limiting comparability. Owing to this pronounced heterogeneity and the diversity of biomarker targets, no meta-analysis was undertaken for mechanistic outcomes.

### 3.12. Gut Microbiota

Across the included trials, several microbiota-directed interventions modulated components of the infant gut microbiota, although effects were heterogeneous across taxa, timepoints, and analytical platforms. Five trials reported Bifidobacteria-related measures using relative abundance, log10 CFU/g counts, or semi-quantitative approaches. In a microbiota-focused Chinese trial [18], supplemented infants showed higher fecal Bifidobacteria median abundance at 16 and 24 weeks compared with controls, while in very preterm infants, van den Berg 2016 documented fluctuating between-group differences across days 1–30 and an exploratory association between higher early Bifidobacteria levels and better MDI scores [21]. In Firmansyah 2011 [27], Bifidobacteria counts and percentages at 12 and 16 months were broadly similar between groups despite modest numerical differences, and Neumer 2021 reported no consistent direction of effect across serial assessments at 2, 6, and 12 months [29].

Lactobacillus or Lactobacillus/Enterococcus clusters were quantified in two studies. Li 2023 observed a significant increase in Lactobacillus relative abundance at week 4 in infants receiving LpN1115 [28], whereas Firmansyah 2011 [27] showed mixed results, with one timepoint demonstrating higher counts and percentages in the intervention arm but others showing no difference, partly influenced by baseline imbalances.

Enterobacteria-related endpoints, reported in Firmansyah 2011 [27] and Neumer 2021 [29], showed no meaningful or consistent differences between groups. Other taxa, including Bacteroides/Prevotella, Clostridia/Eubacteria, *C. coccoides*, *C. leptum*, and total bacterial counts, were assessed in several studies. Van den Berg 2016 [21] reported largely null effects across multiple neonatal timepoints, and both Firmansyah 2011 [27] and Neumer 2021 [29] documented predominantly inconsistent or absent intervention-related differences.

Overall, while some interventions increased presumed beneficial taxa such as Bifidobacteria or Lactobacillus at selected timepoints, the magnitude, consistency, and durability of these effects varied widely, and most other bacterial groups showed no reproducible response to supplementation.

### 3.13. SCFA and Microbial Metabolites

Short-chain fatty acids (SCFAs) were sparsely reported across trials. Only one study quantified fecal butyrate [23], documenting significantly higher concentrations at day 21 in colicky term infants receiving *Bifidobacterium animalis* subsp. *lactis* BB-12 compared with placebo (approximately 0.85 mM vs. 0.55 mM; *p* < 0.0001, extracted from graphical data), suggesting a meaningful probiotic-associated increase in colonic butyrate production.

No other SCFAs, including acetate or propionate, were systematically reported, providing insufficient evidence to determine whether early-life probiotic supplementation broadly alters the SCFA profile beyond butyrate.

### 3.14. Secretory IgA (Fecal and Salivary)

Li 2023 assessed fecal and salivary secretory IgA (sIgA) in C-section-born term infants receiving *Lacticaseibacillus paracasei* N1115 or placebo [28]. Fecal sIgA (mg/g stool) showed a trend toward higher levels in the intervention group and lower levels in the placebo group, with one pre-specified subgroup demonstrating a significant difference in change-from-baseline (intervention +1.12 mg/g vs. placebo −1.02 mg/g). Absolute week-12 means and SDs were not fully reported.

Salivary sIgA (mg/g saliva) declined over time in the placebo group, whereas reductions were less pronounced in probiotic-treated infants, although between-group differences were not clearly significant. Overall, findings suggest a potential enhancement or stabilization of mucosal IgA responses following probiotic supplementation in C-section-born infants, but incomplete reporting limits the strength of inference.

### 3.15. α1-Antitrypsin

Fecal α1-antitrypsin, a marker of intestinal protein loss and permeability, was assessed in Li 2023 at week 12 [28]. Both intervention and placebo groups exhibited small decreases from baseline, with no significant between-group differences in change. These findings indicate that probiotic supplementation did not meaningfully influence fecal α1-antitrypsin levels over the study period.

### 3.16. Antimicrobial Peptides

In Chen 2021 [23], the antimicrobial peptide LL-37 was quantified in stool samples at day 21, with higher mean concentrations in infants receiving *Bifidobacterium animalis* subsp. *lactis* BB-12 compared with controls (approximately 13 ng/g vs. 8 ng/g; *p* = 0.0212, graphically extracted). The same trial measured human β-defensin-2 (HBD-2), which also showed markedly higher levels in the intervention group (around 160 ng/g vs. 80 ng/g; *p* < 0.0001). Taken together, these findings indicate a substantial upregulation of mucosal antimicrobial peptides in colicky infants treated with BB-12, consistent with enhanced innate mucosal defense at the gut level.

### 3.17. Cytokines and Immune Mediators

Cytokine data were reported exclusively in van den Berg 2016 [21], a prebiotic trial in very preterm infants. Circulating concentrations of pro-inflammatory cytokines (IL-1β, IL-6, IL-8, TNF-α), anti-inflammatory IL-10, and Th1/Th2/Th17-related cytokines (IFN-γ, IL-4, IL-17) were measured at birth, day 7, and day 14. Across all timepoints, no consistent between-group differences were observed for IL-1β, IL-6, IL-8, TNF-α, or IL-10, and IL-17 similarly showed no significant intervention effects.

Exploratory analyses suggested associations between higher neonatal IL-8 and TNF-α and lower cognitive performance (MDI), as well as between higher IFN-γ at day 7 and lower motor scores (PDI), indicating potential links between early systemic inflammation and later neurodevelopment, independent of prebiotic allocation. IL-10 showed no clear intervention effect, and exploratory correlations suggesting that lower IL-10 might relate to improved outcomes were inconsistent and non-robust. Overall, prebiotic supplementation did not meaningfully alter systemic cytokine profiles, although the neonatal inflammatory milieu may be related to subsequent neurodevelopmental trajectories.

### 3.18. Intestinal Inflammation, Permeability and Gut Environment

Fecal calprotectin was reported in three trials [23,25,28]. In colicky infants [23], mean calprotectin concentrations at day 21 were modestly higher in the probiotic group (≈900 mg/kg) than in controls (≈700 mg/kg; *p* = 0.0422). Nocerino 2020 [25] reported baseline values that were broadly similar between groups (≈550–600 mg/kg), with slightly greater increases at follow-up in the intervention arm, although differences were small and partly derived from graphical extraction. In contrast, Li 2023 [28] observed similar changes in calprotectin across groups over 12 weeks, with no significant between-group differences.

Overall, calprotectin responses were heterogeneous, with small increases in some trials and null effects in others, and no clear pattern linking these changes to neurodevelopmental outcomes. Fecal pH was evaluated in Li 2023 [28]. By week 12, the control group exhibited a significant rise in fecal pH, whereas pH in the probiotic group remained relatively stable (reported control mean 6.34), suggesting a modest attenuation of alkalinization with *L. paracasei* N1115. Beyond α1-antitrypsin, no additional permeability markers were reported; fecal α1-antitrypsin itself showed no significant between-group differences, indicating no measurable effect of probiotic supplementation on gross intestinal protein loss or permeability.

### 3.19. Stress Physiology/HPA Axis

Salivary cortisol was assessed in Li 2023 at baseline and week 12 [28]. Cortisol levels decreased from baseline in the probiotic group, whereas changes in the placebo group were less pronounced; however, only change-from-baseline values were provided, and between-group differences at week 12 were not statistically significant. These results tentatively suggest a potential attenuation of HPA axis activity in C-section-born infants receiving *L. paracasei* N1115, although evidence is limited to a single trial with incomplete reporting

### 3.20. Integrated Synthesis of Mechanistic Outcomes

Across the eight trials reporting mechanistic endpoints, microbiota-targeted interventions produced diverse but heterogeneous effects on the gut ecosystem and host physiological responses.

Several studies demonstrated increases in Bifidobacteria or Lactobacillus/Enterococcus abundance, whereas effects on Enterobacteria, Bacteroides/Prevotella, Clostridial groups, and total bacterial load were inconsistent. Only one preterm study reported exploratory correlations between higher early Bifidobacteria abundance and better MDI scores. SCFA data were limited to one colic trial, which showed a clear increase in fecal butyrate with BB-12 supplementation, with no comparable data for acetate or propionate. Mucosal immune readouts showed modest increases in fecal or salivary sIgA in C-section-born infants and robust elevations in antimicrobial peptides (LL-37 and HBD-2) in colicky infants receiving BB-12, suggesting enhanced innate mucosal activity.

Systemic cytokine profiles in very preterm infants were not altered by prebiotic supplementation, although higher neonatal IL-8, TNF-α, and IFN-γ were associated with poorer neurodevelopment in exploratory analyses, independent of treatment assignment. Markers of intestinal inflammation and permeability showed variable responses: calprotectin exhibited small increases in some studies and no differences in others; α1-antitrypsin showed no measurable effect; and fecal pH demonstrated a modest attenuation of alkalinisation with *L. paracasei* N1115. Stress-axis data were limited to a single study, suggesting reduced salivary cortisol in the intervention group.

Overall, mechanistic outcomes indicate that early probiotic or microbiota-directed interventions can modulate gut microbial composition, SCFA production, mucosal immunity, and antimicrobial peptide expression. However, these biological shifts were highly heterogeneous, often reported with incomplete numerical detail, and did not translate into consistent improvements in neurodevelopmental outcomes, precluding the identification of a reproducible mechanistic pathway. Importantly, although several studies reported changes in microbial taxa or microbial metabolites such as short-chain fatty acids, these biological signals were not consistently linked to measurable neurodevelopmental improvements across trials. This may reflect the complexity of the gut–brain axis and the possibility that microbial changes alone are insufficient to influence neurodevelopmental trajectories without additional environmental or host factors. 

## 4. Discussion

This systematic review and meta-analysis examined whether early-life microbiota-directed interventions, including probiotics, prebiotics, and synbiotics, are associated with improved neurodevelopmental outcomes in infants aged 0–36 months. Across the available randomized controlled trials and within the limits of the current evidence base, we found no convincing evidence that these interventions enhance cognitive, language, or motor development when assessed using validated standardized instruments. These findings are consistent with recent meta-analyses reporting largely null or inconsistent effects of probiotic supplementation on early neurodevelopment [30]. Despite strong biological plausibility supporting gut–brain interactions during critical periods of early development, our findings suggest that postnatal modulation of the infant gut microbiota, as implemented in existing trials, is insufficient to generate clinically meaningful neurodevelopmental gains.

Experimental studies demonstrate that early microbial perturbations can influence neural maturation, synaptogenesis, stress responsivity, myelination, and social behavior [31,32], while observational studies in infants have reported associations between microbiota composition and cognitive or socio-emotional outcomes [4,5]. However, translating these mechanistic and associative observations into reproducible clinical benefits has proven challenging. Our results align with a recent systematic review including more than 3000 infants, which reported no overall cognitive advantage associated with probiotic supplementation, with the exception of a modest signal in trials with intervention durations exceeding six months [30].

This observation supports the hypothesis that timing and duration of exposure may be important moderators of potential gut–brain effects. Given the rapid and dynamic nature of neurodevelopment during infancy, relatively brief supplementation periods, common across infant RCTs, may be insufficient to influence longer-term developmental trajectories.

Evidence from preterm populations was similarly heterogeneous. While certain probiotic strains, such as Bifidobacterium breve M-16V or multi-strain formulations, have demonstrated benefits in reducing late-onset sepsis or necrotizing enterocolitis, large meta-analyses and long-term follow-up studies have consistently shown no improvement in cognitive or motor outcomes at 18–24 months [33,34]. Although isolated studies have suggested potential neurodevelopmental benefits in extremely preterm infants, these findings have not been consistently replicated and may reflect population-specific vulnerabilities, residual confounding, or methodological limitations rather than robust treatment effects [35]. Importantly, most included trials reported measurable biological effects of microbiota-directed interventions, including increases in Bifidobacteria or Lactobacillus abundance and changes in microbial metabolites such as short-chain fatty acids. However, these biological shifts were not associated with parallel improvements in neurodevelopmental outcomes.

This dissociation may reflect redundancy within gut–brain signaling pathways, insufficient magnitude or durability of microbial modulation, or developmental buffering mechanisms that limit the impact of postnatal microbial changes on higher-order neurodevelopmental outcomes. Alternatively, neuroactive pathways influenced by probiotics, such as immune signaling, microbial metabolite production, or vagal modulation, may require earlier, more sustained, or more targeted interventions than those evaluated in current RCTs.

Additional factors likely contributing to the absence of detectable neurodevelopmental effects include substantial heterogeneity in probiotic strains, dosages, and formulations; limited sample sizes; relatively short follow-up periods; and reliance on global developmental instruments, which may lack sensitivity to subtle or domain-specific effects. 

This clinical heterogeneity should be considered when interpreting pooled estimates, as differences in infant populations (term versus preterm infants), intervention composition, and treatment duration may influence treatment responsiveness and limit the comparability of studies included in the meta-analysis. The included trials used a wide variety of probiotic strains, dosages, and intervention durations. This heterogeneity may obscure potential strain-specific effects, as different microbial strains can exert distinct biological and immunomodulatory properties. Strain-specific subgroup analyses were considered; however, the limited number of available trials and the diversity of interventions prevented meaningful subgroup comparisons. Furthermore, several trials enrolled infants with functional gastrointestinal conditions such as colic or formula intolerance, in whom symptomatic improvement may primarily affect parental well-being rather than objectively measurable neurodevelopmental outcomes, a pattern consistent with our findings.

Overall, this synthesis indicates that the current body of evidence does not support the use of early-life probiotics, prebiotics, or synbiotics for the purpose of enhancing neurodevelopment in either healthy term infants or preterm populations. Nevertheless, the modest signals observed in longer-duration trials, together with compelling mechanistic rationale, highlight the need for future studies that are strain-specific, adequately powered, and designed with extended follow-up beyond 24 months.

Such trials should integrate harmonized microbiome, metabolomic, and immunologic profiling and prioritize high-risk subgroups, responder phenotypes, and outcome measures capable of capturing subtle gut–brain effects, including neurophysiological or imaging-based markers. In conclusion, while early microbiota modulation remains a theoretically promising pathway for influencing brain development, current clinical evidence does not demonstrate a consistent neurodevelopmental benefit of probiotic, prebiotic, or synbiotic supplementation in infancy. More rigorous, mechanistically anchored, and longitudinal studies are required before these interventions can be recommended for neurodevelopmental enhancement. Several additional factors may influence the relationship between microbiota modulation and neurodevelopmental outcomes. Variables such as breastfeeding status, early-life antibiotic exposure, and mode of delivery are known to shape the developing infant microbiome and may act as important confounding factors in studies evaluating microbiota-targeted interventions. Differences in these variables across trials may therefore contribute to variability in microbial responses and potentially influence downstream neurodevelopmental trajectories.

## 5. Limitations

This systematic review and meta-analysis should be interpreted in light of several limitations. In addition, several included trials had relatively small sample sizes, which may have limited the statistical power to detect modest neurodevelopmental effects. The available evidence base remains limited, as relatively few randomized controlled trials have evaluated validated neurodevelopmental outcomes following early-life microbiota-targeted interventions. Only a subset of studies reported sufficiently homogeneous measures, primarily Bayley-III composite scores, to allow quantitative synthesis, which reduced the precision of pooled estimates and precluded formal exploration of potential moderators such as strain specificity, dosage, or timing of supplementation. Substantial clinical and methodological heterogeneity was evident across studies, including differences in microbial formulations, intervention duration, and neurodevelopmental assessment tools applied at varying ages.

Follow-up periods were generally short, with most outcomes assessed before two years of age, limiting the ability to detect later-emerging cognitive or socio-emotional effects. In addition, reliance on global developmental instruments may have reduced sensitivity to subtle or domain-specific neurodevelopmental differences.

Mechanistic outcomes were reported inconsistently and varied widely in analytic methods, units of measurement, and sampling timepoints, preventing meaningful synthesis and limiting inference regarding causal pathways linking microbiota modulation to neurodevelopment. Incomplete outcome reporting in several trials, including missing variance estimates or graphical-only data, required digital extraction and may have introduced additional imprecision. Missing outcome data and loss to follow-up were not uniformly addressed.

Finally, generalizability is limited, as most trials were conducted in healthy term infants or infants with mild functional conditions, while evidence in higher-risk populations remains sparse. Publication bias cannot be formally excluded given the small number of studies and the inability to assess small-study effects.

## 6. Conclusions

This systematic review and meta-analysis show that current evidence from randomized controlled trials does not support a clinically meaningful benefit of early-life probiotic, prebiotic, or synbiotic supplementation on neurodevelopmental outcomes during the first three years of life. Across validated standardized assessments, no consistent differences were observed between intervention and control groups, and biologically detectable changes in gut microbiota composition or immune markers did not translate into improved neurodevelopmental trajectories. These findings indicate that, as implemented in existing trials, microbiota-targeted interventions are insufficient to meaningfully influence early brain development in either healthy term infants or preterm populations.

Routine use of probiotics, prebiotics, or synbiotics for the purpose of enhancing neurodevelopment cannot therefore be recommended. Future research should focus on rigorously designed, adequately powered, strain-specific trials with standardized developmental assessments and extended follow-up into later childhood, particularly in high-risk populations, to determine whether targeted microbiota modulation may yet confer neurodevelopmental benefit.

## Data Availability

No new data were created in this study. Data supporting the findings of this study are available within the article and its references.

## Supplementary Materials

The following supporting information can be downloaded at: https://www.mdpi.com/article/10.3390/cells15070638/s1, Table S1: PRISMA 2020 checklist.

## Abbreviations

The following abbreviations are used in this manuscript:

ASQ	Ages and Stages Questionnaire
Bayley-III	Bayley Scales of Infant Development, Third Edition
BSID-II	Bayley Scales of Infant Development, Second Edition
CI	Confidence Interval
CFU	Colony Forming Units
GRADE	Grading of Recommendations Assessment, Development and Evaluation
HPA	Hypothalamic–Pituitary–Adrenal
IQR	Interquartile Range
MD	Mean Difference
MSEL	Mullen Scales of Early Learning
PRISMA	Preferred Reporting Items for Systematic Reviews and Meta-Analyses
RCT	Randomized Controlled Trial
SCFA	Short-Chain Fatty Acids
sIgA	Secretory Immunoglobulin A
