# Supplementary material for: Early-Life Microbiota Modulation and Neurodevelopment in Infants: A Systematic Review and Meta-Analysis of Randomized Controlled Trials
Source: Cells. 2026 Apr 1;15(7):638. doi: 10.3390/cells15070638 (PMC13072312; doi:10.3390/cells15070638)
Supplement: Supplementary file 1 [file cells-15-00638-s001.zip › cells-4212524-supplementary.pdf]

Supplementary Material S1 – PRISMA 2020 Checklist

Section	Item	Checklist item	Location in manuscript
Title	1	Identify the report as a systematic review	Title
Abstract	2	Structured summary	Abstract
Introduction	3	Rationale	Introduction
Introduction	4	Objectives	Introduction (end paragraph)
Methods	5	Eligibility criteria	Materials and Methods – 2.2 Eligibility criteria
Methods	6	Information sources	Materials and Methods – 2.7 Information sources
Methods	7	Search strategy	Materials and Methods – 2.7 Search strategy
Methods	8	Selection process	Materials and Methods – 2.8 Study selection
Methods	9	Data collection process	Materials and Methods – 2.9 Data extraction
Methods	10	Data items	Materials and Methods – 2.9 Data extraction
Methods	11	Risk of bias assessment	Materials and Methods – Risk of bias section
Methods	12	Effect measures	Materials and Methods – Statistical analysis

Methods	13	Synthesis methods	Materials and Methods – Statistical analysis
Methods	14	Reporting bias assessment	Materials and Methods – Statistical analysis
Methods	15	Certainty assessment	Materials and Methods – Risk of bias / GRADE
Results	16	Study selection	Results + Figure 2 (PRISMA flow diagram)
Results	17	Study characteristics	Results – Study characteristics
Results	18	Risk of bias in studies	Results – Risk of bias assessment
Results	19	Results of individual studies	Results – Individual study results
Results	20	Results of syntheses	Results – Meta-analysis results
Results	21	Reporting biases	Results – Publication bias assessment
Results	22	Certainty of evidence	Results – Evidence certainty
Discussion	23	Discussion	Discussion
Other information	24	Registration and protocol	Materials and Methods – Study design and registration (PROSPERO)
Other information	25	Support	Funding
Other information	26	Competing interests	Conflicts of Interest
Other information	27	Availability of data, code and materials	Data Availability Statement

From: Page MJ, McKenzie JE, Bossuyt PM, Boutron I, Hoffmann TC, Mulrow CD, et al. The PRISMA 2020 statement: an updated guideline for reporting systematic reviews. BMJ 2021;372:n71. doi: 10.1136/bmj.n71. This work is licensed under CC BY 4.0. To view a copy of this license, visit https://creativecommons.org/licenses/by/4.0/
